# How do naloxone-based interventions work to reduce overdose deaths: a realist review

**DOI:** 10.1186/s12954-022-00599-4

**Published:** 2022-02-23

**Authors:** Nicole M. Miller, Bethany Waterhouse-Bradley, Claire Campbell, Gillian W. Shorter

**Affiliations:** 1 Institute of Mental Health Sciences, School of Psychology, Ulster University, Coleraine, UK; 2 School of Applied Social and Policy Sciences, Ulster University, Belfast, UK; 3 School of Psychology, Ulster University, Coleraine, UK; 4 Drug and Alcohol Research Network & Centre for Improving Health Related Quality of Life, School of Psychology, Queen’s University Belfast, Belfast, UK

**Keywords:** Naloxone, Realist review, Bystander response, Opioid overdose, Drug-related deaths

## Abstract

**Background:**

Naloxone-based interventions as part of health systems can reverse an opioid overdose. Previous systematic reviews have identified the effectiveness of naloxone; however, the role of context and mechanisms for its use has not been explored. This realist systematic review aims to identify a theory of how naloxone works based on the contexts and mechanisms that contribute to the success of the intervention for improved outcomes.

**Methods:**

Pre-registered at PROSPERO, this realist review followed RAMESES standards of reporting. Keywords included 'naloxone' and ' opioid overdose'. All study designs were included. Data extraction using 55 relevant outputs based on realist logic produced evidence of two middle-range theories: Naloxone Bystander Intervention Theory and Skills Transfer Theory.

**Results:**

Harm reduction and/or low threshold contexts provide a non-judgemental approach which support in-group norms of helping and empower the social identity of the trained and untrained bystander. This context also creates the conditions necessary for skills transfer and diffusion of the intervention into social networks. Stigma and negative attitudes held by first responders and stakeholders involved in the implementation process, such as police or GPs, can prohibit the bystander response by inducing fear in responding. This interferes with skills transfer, naloxone use and carriage of naloxone kits.

**Conclusions:**

The findings provide theoretically informed guidance regarding the harm reduction contexts that are essential for the successful implementation of naloxone-based interventions. Peer-to-peer models of training are helpful as it reinforces social identity and successful skills transfer between bystanders. Health systems may want to assess the prevalence of, and take steps to reduce opioid-related stigma with key stakeholders in contexts using a low threshold training approach to build an environment  to support positive naloxone outcomes.

***Trial Registration*:**

PROSPERO 2019 CRD42019141003.

**Supplementary Information:**

The online version contains supplementary material available at 10.1186/s12954-022-00599-4.

## Contributions to the literature

- Previous research on effectiveness of naloxone-based interventions has not explored how the context surrounding the intervention relates to outcomes.
- This realist review provides a novel programme theory by integrating bystander effects, social identity theory, and skills training with the contexts and mechanisms that make this training successful for the reduction of opioid overdose deaths.
- The resulting theories further our understanding of how low threshold training design supports social identity and in-group norms (of people who use drugs), which supports the conditions for the success of a peer-to-peer distribution model of naloxone. We highlight how stigma can interfere with this process.

## Background

Opioid overdose is a global public health issue [1]. Take-home naloxone, a common overdose prevention intervention, is an opioid agonist given  by intranasal or by intramuscular injection to reverse the effects of opioid poisoning. It is a proven strategy to reverse an overdose when combined with training in overdose management [2, 3]. Complex interventions, like naloxone, require an evaluation of the intervention along with its context (policy and social environment) to understand how it produces its intended or unintended outcomes [4]. The contextual factors that enable or create barriers to naloxone use in curbing overdose deaths are unknown. Understanding this relationship can inform the design and implementation of naloxone-based intervention programmes to be more effective in reducing opioid overdose deaths.

### Rationale and objectives for the review

Systematic reviews which establish the effectiveness of naloxone in preventing deaths from opioid overdose describe the training contexts and the motivations to use naloxone. However, they do not describe how these factors relate to outcomes [5–7]. Several systematic reviews on naloxone training in a community-based context have outlined that willingness to help and retention of training knowledge are factors that facilitate naloxone use. Fear of arrest is a barrier in training with this context [7, 8]. However, these reviews do not show an understanding of why a community-based context motivates naloxone use and barriers to outcomes [7, 8]. In other systematic reviews, effective trainees (those who use needle exchange services, or opioid detoxification patients, and/or family and friends of people who inject drugs) for naloxone-based programmes are established. How the training context relates to successful skills transfer in an overdose is unknown [6–8]. On the opposite spectrum, the moral hazard model attempts to explain opioid mortality outcomes by suggesting that increased access to naloxone motivates opioid use leads to increase opioid-related deaths [9]. This research lacks an explanation of what contextual factors enable such outcomes. This points to a gap in our understanding of how the context of naloxone training relates to outcomes [9, 10].

### Rationale for a realist review

A realist review method explains how the interventions work through understanding of the relationship between an intervention context and the mechanisms (barriers or facilitators) that lead to intervention outcomes [11–13]. The realist review method can identify how context relates to the use of naloxone-based strategies—as defined as strategies where naloxone is used as a part of the intervention—to prevent overdose deaths. This review answers the following questions: What context results in the use of naloxone-based interventions in an opioid overdose? What are the key mechanisms that characterise naloxone-based intervention use or non-use?

## Methods

This review followed RAMESES reporting standards for realist reviews (see Additional file 1 for the RAMESES checklist) [11]. The review occurred in four stages as (1) scope and initial theory development, (2) literature search and theory refinement, (3) literature synthesis resulting in a final theory, and (4) presentation of the study characteristics and evidence of the final theory of how naloxone-based interventions work to prevent opioid overdose.

- **Stage one: scope and initial theory development**

An initial theory explaining how naloxone-based interventions worked was developed through a scope of the literature. Backwards citation of systematic reviews on the use of naloxone to reduce opioid overdose occurred first [6, 8]. We reviewed this literature for theories that explicitly described how naloxone worked to prevent overdose. When no explicit theory was present, a search for a substantive theory occurred. In addition, a theory based on realist logic was developed using hypothetical 'if-then' statements. The statements described the if - under what circumstances did the intervention have then - certain outcomes. A heuristic called context, mechanism, and outcome configuration (CMOc) was used to outline the evidence of the initial theory. Contexts that depicted institutional, infrastructural, interpersonal relationships and individuals making up the backdrop of the programme were noted [12]. Mechanisms were categorised by behaviour and/or thoughts generated within the target group of people who use opioids that motivated the use of the intervention. This behaviour shaped expected (intended) or not expected (unintended) outcomes and informed how the intervention worked in practice [11, 13]. The scope resulted in two initial programme theories using 13 relevant studies [2, 14–25]. NMM developed the two initial theories.

### Initial theory one: Naloxone Bystander Intervention Theory (NBIT)

The substantive theories of bystander effects, social identity, and self-categorisation were integrated and  informed the first programme theory, Naloxone Bystander Intervention Theory (NBIT). Three sub-theories were developed from this theory as: NBIT-A, NBIT-B, and NBIT-C. Individual response to emergencies can be delayed when in the presence of a group otherwise known as bystander effects [26]. Bystander effects are limited, however, when the person who needs help shares the same social category and/or same social identity as the bystander. This in-group favouritism leads to quicker responses [27].

Many of the studies described training in low threshold or harm reduction settings. A low threshold context for this review was interchangeable with harm reduction. It was defined as contexts that hold non-judgemental attitudes, respect, and belief in a person’s right to use drugs, with aims to protect health, and reduce stigma [14, 18, 22]. Low threshold contexts supported the social identity of a person who uses opioids to take part in the naloxone-based interventions [18]. Such a context generated a feeling of acceptance (mechanism) of being a person who uses drugs (social identity). This then led to accepting other group members as being worthy of help, enhancing in-group norms. This generated willingness (mechanism) to help someone within their shared social category and a faster bystander response [14]. The intervention 'worked' as evidenced by reports of reversals being performed on a peer/someone within their social network by a trained bystander (NBIT-A) [24].

A low threshold context had a spill over effect as exhibited by reports of trained bystanders that members of their social networks not trained to use naloxone reversed an overdose (outcome) (NBIT-B) [3]. The mechanism of willingness motivated the use of naloxone by a bystander that was untrained. There was also a third context. Negative attitudes (out-group norms) towards people who use drugs—in this case a trained bystander—by stakeholders (e.g. probation officers and first responders such as police) alongside a low threshold training generated different mechanisms [14, 23]. There was evidence of fear and feelings of being stigmatised in the potential bystander generated from this context, which appeared to interfere with the use of naloxone (NBIT-C). This suggested that bystander effects would increase. For example, a trained bystander may encounter someone who is overdosing with whom they share the same social identity; however, fear of negative consequences (i.e. police arrest) associated with helping hindered the response to help [14].

### Initial theory two: Skills Transfer Theory (STT)

A second theory of how naloxone-based interventions work was based on a Skills Transfer Theory (STT). This theory had two sub-theories: STT-A and STT-B. This theory emphasised supplying skills in overdose management to a person who uses opioids to respond to an opioid overdose [2, 16]. Skills training was provided by a counsellor or member of the programme staff or through a Train the Trainer delivery method [2, 14–16, 21–23]. Skills training and Train the Trainer models further refined the theory [28–30]. A blend of relevant training, trust, and the need for using the skills facilitated the environment necessary for skills transfer to reverse an overdose.

The contexts of a non-judgemental attitude within a low threshold environment generated trust (mechanism) in the skills being learned, and self-efficacy (mechanism) regarding their ability to manage an overdose. This led to outcomes where trainees responded and successfully reversed an overdose using naloxone-based interventions (STT-A) [24]. Contexts where naloxone training was unsuccessful involved training groups of people who were abstinent (former), and people who use opioids. This context appeared to generate a lack of willingness and motivation to use naloxone. The trainee may not use the skills because they are no longer using drugs and may not feel they will be exposed to opioid overdoses through their networks (unintended outcome) (STT-B) [23].

Stakeholder consultations (*T* = 5) occurred with providers of overdose prevention services from Northern Ireland (location of the authors of this review). Analysis of CMOc from the discussions refined the theory. The dialogue with the stakeholders confirmed a low threshold context would generate self-efficacy, responsibility, and empowerment. This motivated the service user to help in an overdose situation (outcome). This further refined the theory and members of the research team (NMM, BWB, CC, and GWS) agreed on the candidate theory for the second stage of the review.

- **Stage two: literature search and theory refinement**

**Fig. 1:**
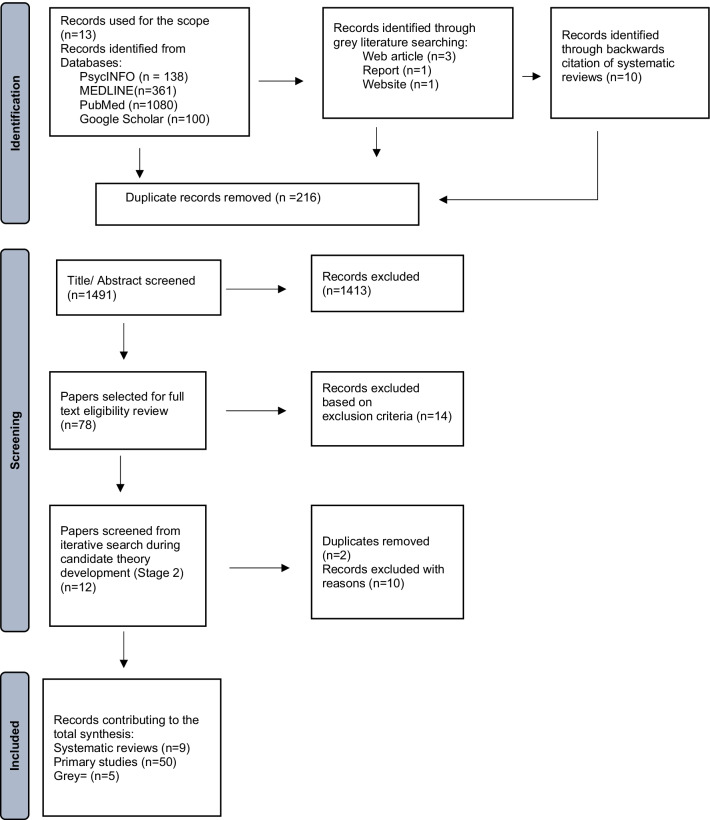
Flow diagram illustrating stage 2 search process for the realist review of naloxone-based interventions

The second stage of this review refined the candidate theory using a systematic search of the literature using databases PsycINFO, MEDLINE, PubMED and Google Scholar. Key search terms as “naloxone or Narcan”, AND “opioids or opiates”, AND “overdose prevention” were used. A search of the same terms was placed into Google Scholar for the time frame 1996–2019. However, only the first 100 items from Google search were screened because of the limited time to complete the realist review. Backwards citation tracking using systematic reviews on naloxone occurred to identify new citations [5, 7, 31–35]. A search for grey literature included public health documents from the Republic of Ireland and Northern Ireland, United States of America, and Estonia [36–39]. Figure 1 summarises the search process.

Identification and selection tools based on the candidate theory were developed to guide the screening and eligibility criteria of the review (see Additional file 2 Identification and selection tools for the realist review of naloxone-based interventions 2). Records given a full text reading were appraised based on the relevance of the paper to support the theory. Rigour was evident by the consistency between methods used for the studies and their outcomes (see Additional file 3 Appraisal and rigor tools for the realist review of naloxone-based interventions 3 ) [13]. Characteristics of the studies were inputted into an excel database, followed by data extraction of CMOc to refine the theory (see Additional file 4 Data extraction and synthesis tools for the realist review of naloxone-based interventions for examples of data extraction). Saturation became evident when there was no additional evidence of CMOc and the review of literature ended.

- **Stage three: literature synthesis**

The candidate theory was revised into a final theory by mapping out a semi-predictable pattern of the CMOc found in the second stage of the review. The mapping out process allowed easy categorisation of the CMOc and the CMOc were grouped into demi-regularities. These demi-regularities become the middle-range theory that informed how the intervention worked to specific and various contexts (see Additional file 4 Data extraction and synthesis tools for the realist review of naloxone-based interventions 4 for the analytical process of the synthesis).

The diffusion of innovation (DOI) theory helped refine the NBIT-B candidate theory [40, 41]. It supplied an understanding of the mechanisms and outcomes which generated reports of the use of naloxone by an untrained bystander. An iterative search using new terms “naloxone” and “diffusion of innovation theory” (DOI) with databases of PsycINFO, MEDLINE, PubMed took place. There were no citations found for PsycINFO and MEDLINE. There were 12 new items found in PubMed out of the 12 items, two duplicates were found, and the remaining did not fit the inclusion criteria (included in Fig. 1).

The DOI theory refined the understanding of why an untrained bystander would use the intervention and how it becomes distributed within social networks of people at risk for overdose. For example, a bystander may witness the administration of naloxone and then share the intervention (mechanism) with others as a method of preventing overdose. The ripple effect, whereas the context and mechanisms generated in the first CMOc created a new CMOc, was found here [42]. This was evident in the production of a second context of a peer-to-peer training (target group to those not trained). This led to the adoption of naloxone as an innovation (mechanism) and diffusion of it into the social networks. This social diffusion also implies a group norm response to helping using naloxone—another aspect of in-group favouritism and emergency response [27]. This explained why trained bystanders reported witnessing others using the naloxone in their community (outcome) [41].

An additional context further refined the STT. The context of low threshold environments generated empowerment, use of overdose management skills, and administration of naloxone by the trainee (mechanism). Outcomes such as lack of post overdose management, namely calling an ambulance, occurred in this chain [22]. The experience of empowerment appeared to guide the desicion to not use emergency help and explains why trainees did not call for ambulance support. There is a risk of overdosing again despite successful administration of naloxone, but these outcomes were not reported.

These other demi-regularities were added to a middle-range theory that informed how the intervention worked in both specific and various contexts. There was limited evidence for candidate theory STT-B (described in the scope stage) and the theory was not kept in the final synthesis due to not fitting the requirements for relevance (see Additional file 3 Appraisal and rigor tools for the realist review of naloxone-based interventions 3). NMM searched, extracted, and synthesised the data from the literature. BWB and GWS independently reviewed and verified 10% of the papers used for selection and the appraisal stages of the review. All members of the research team (NMM, BWB, CC, and GWS) discussed and agreed upon the final theory. Resolution of discrepancies was by discussion.

## Results

- **Stage four: study characteristics and evidence of the final theory**

### Study characteristics

There were six systematic (narrative) reviews, one mapping review, one scoping review and one systematic review that also had a meta-analysis [5–8, 31–35]. Systematic reviews were used for citation tracking only and 55 records were used for theory building. There were 29 quantitative pieces of scientific literature, 14 qualitative, seven mixed methods and five pieces of grey literature (e.g. news articles, reports) (see Table 1 of included studies and their design. Additional file 5 References for the realist review of naloxone-based interventions 5 contains references for the studies used in these reviews). From these studies, 30 discussed the use of intramuscular administration of naloxone, 12 did not mention a type of naloxone, eight used intranasal, and five used both intranasal and injection methods. The method of training delivery varied. There were 17 studies that used a combination of groups and one-to-one training, six studies used a practice component, three had didactic sessions, two had groups only, two had one-to-one only, and two had video instruction. There were 23 outputs that did not state the method of delivery.

**Table 1:** Summary of included studies (*n* = 50), grey literature (*n* = 5) and how they contributed to the final theories

Full reference	Study design	Programme context	Contribution to synthesis				
			NBIT-A	NBIT-B	NBIT-C	ST-A	ST-B
Ambrose et al., 2016^1^	Bivariate and multivariate associations, sensitivity analysis	Harm reduction programme	X	X			
Baca et al., 2007^2^	Qualitative structured interviews	Harm reduction programme, syringe exchange (low threshold)					X
Banjo et al., 2014^3^	Evaluation (mixed methods)	BCCD harm reduction programme	X	X	X	X	X
Behar et al., 2015^4^	Survey (repeated measures)	DOPE programme (low threshold), syringe exchange (low threshold)	X			X	
Bennett et al., 2011^5^	Evaluation (survey, repeated measures)	Harm reduction programme Negative attitudes (police)	X		X	X	X
Bennett& Holloway, 2012^6^	Evaluation (survey, repeated measures)	Harm reduction programme	X				
Beswick et al., 2002^7^	Qualitative structured interviews	Unknown				X	
Bowles et al., 2019^8^	Qualitative semi structured interviews	Harm reduction programme, syringe exchange (low threshold)	X	X			
Clark & Eustace, 2016^9^	Evaluation (mixed methods-repeated measures and semi-structured interviews)	Harm reduction programme Negative attitudes (General Practitioner [GP])			X		
Chronister et al., 2018^10^	Evaluation (survey repeated measures)	Harm reduction, low threshold health care facility and abstinence-based programmes where low threshold services are embedded	X	X		X	
Das et al., 2017^11^	Case study	Needle exchange (low threshold)		X			
Dettmer et al., 2001^12^	Case study	Health care project, mobile drug services (low threshold)	X			X	
Doe-Simkins et al., 2009^13^	Evaluation (prospective cohort)	Needle exchange (low threshold)	X	X		X	X
Doe- Simkins et al., 2014^14^	Retrospective cohort study	Syringe exchange (low threshold)	X	X		X	X
Dwyer et al., 2015^15^	Evaluation (survey repeated measures)	Low threshold, detoxification centres, abstinence-based residential drug treatment	X	X		X	
Enteen et al., 2010^16^	Evaluation (prospective cohort)	DOPE programme (low threshold), syringe exchange (low threshold) opioid substitution clinic, hostels	X	X			X
Espelt et al., 2017^17^	Survey (repeated measures)	Needle exchange (low threshold), outreach, supervised injection facilities (harm reduction)	X			X	
EuroNPUD, 2019^18^	Report (grey literature)	Unknown	X	X		X	
Galea et al., 2006^19^	Pilot (pre-/post-overdose experiences)	Syringe exchange (low threshold)	X			X	
Gaston et al., 2009^20^	Evaluation (prospective cohort, survey repeated measures)	Abstinent-based programme (inpatient and outpatient) Negative attitudes (police)			X		
George et al., 2010^21^	Case study	Community drug team	X			X	
Gilbert et al., 2018^22^	Randomised controlled trial	SKOOP training programme (harm reduction)	X			X	
Green et al., 2008^23^	Between groups (trained vs untrained, questionnaire)	Needle exchange (low threshold) and abstinence-based drug treatment centre	X				
Green et al., 2014^24^	Case study	Prison naloxone programme	X	X		X	
Farrugia et al., 2019^25^	Case study	Peer run drug consumer organisation		X		X	
Khatiwoda et al., 2018^26^	Mixed methods (survey and open-ended questions)	Harm reduction	X			X	
Lankenau et al., 2013^27^	Evaluation qualitative-closed and open-ended questions)	Syringe exchange (low threshold), community health care programmes Negative attitudes (police)	X	X	X	X	X
Leece et al., 2013^28^	Evaluation (cohort)	Syringe exchange (low threshold)	X			X	
Leece et al., 2016^29^	Evaluation (mixed methods-survey and one-to-one interviews)	Syringe exchange (low threshold)	X	X		X	
Madah-Amiri, et al., 2017^30^	Evaluation (prospective cohort, overdose prevention behaviours)	Syringe exchange, supervised injection site (low threshold)	X	X		X	
Maldah-Amiri, et al., 2019^31^	Prospective cohort (survey, repeated measures)	Syringe exchange (low threshold)	X			X	
Maldjian, et al., 2016^32^	Prospective cohort (survey, repeated measures)	Syringe exchange (low threshold)	X			X	
Maxwell (2006)^33^	Report (prospective cohort, post training knowledge)	Chicago Recovery Alliance programme (harm reduction)	X				
McAuley et al., 2010^34^	Evaluation (prospective cohort, post training knowledge)	Lanarkshire naloxone project (low threshold)	X				
Nelson et al., 2016^35^	Evaluation (mixed methods, survey repeated measures, one-to-one interviews)	Low threshold	X	X		X	X
NIHD (n.d.)^36^	Report (post training actions taken in an overdose)	Harm reduction	X			X	
Olsen et al., 2015^37^	Evaluation (mixed methods-survey repeated measures, semi-structured interviews)	Harm reduction	X			X	X
PHA, 2016^38^	Report (post training)	Low threshold services	X			X	X
Parmar et al., 2017^39^	Randomised control pilot trial	N-Alive programme (low threshold)				X	
Piper et al., 2008^40^	Evaluation (survey)	SKOOP programme (harm reduction)	X				
Rowe et al., 2015^41^	Survey	Harm reduction	X			X	
Ruane, 2019^42^	News article (describing barriers to naloxone programmes)	Harm reduction Negative attitudes (GP)			X		
Seal et al., 2005^43^	Pilot study (prospective cohort, survey)	Harm reduction Negative attitudes (Police)	X		X	X	X
Sherman et al., 2008^44^	Qualitative one-to-one semi-structured interviews	Harm reduction centre, syringe exchange (low threshold) Negative attitudes (ambulatory staff)	X		X	X	
Sherman et al., 2009^45^	Qualitative one-to-one interviews	Staying Alive programme (low threshold), needle exchange (low threshold)	X	X		X	
Shorter & Bingham, 2016^46^	Evaluation (qualitative semi structured interviews)	Negative attitudes (police)			X		
Siegler et al., 2017^47^	Prospective cohort (questionnaire)	Syringe exchange programme (low threshold)	X			X	
Strang et al., 2008^48^	Prospect cohort (pre-/post-training questionnaire)	Abstinence-based inpatient and outpatient drug treatment programme	X				
Tobin et al., 2009^49^	Evaluation (prospective cohort, pre-/post-training)	Staying Alive programme (harm reduction), syringe exchange (low threshold)	X			X	X
Traynor, 2019^50^	News article (reports of actions taken in an overdose)	Harm reduction and community outreach programmes	X			X	
Wagner et al., 2010^51^	Evaluation (questionnaire)	Harm reduction	X	X		X	
Wagner et al., 2014^52^	Qualitative one-to-one interviews	Syringe exchange within a community health care, Homeless Health Care, Los Angeles centre for harm Reduction, Skid Row Negative attitudes (police)	X		X	X	
Walley et al., 2013^53^	Interrupted time series analysis	Harm reduction, Chicago Recovery Alliance programme	X		X		
Worthington et al., 2006^54^	Qualitative focus group interviews	Lower east side harm reduction centre Negative attitudes (police)	X		X		
Yokell et al., 2011^55^	Evaluation (post training)	Mixed: syringe exchange, AIDS outreach, homeless shelters, abstinence-based substance abuse treatment programmes				X	

## Evidence of the final theory

There were two final theories with associated sub-theories. Table 1 outlines all retained outputs with their associated programme theory.

### Programme theory 1: Naloxone Bystander Intervention Theory

There were three sub-theories for Naloxone Bystander Intervention Theory as NBIT-A, NBIT-B, and NBIT-C. There were two sub-theories that outlined how a low threshold or harm reduction context can benefit naloxone-based intervention training outcomes. This context appeared to minimise bystander effects based on the victim and trainees’ shared social category/network. Contexts where negative attitudes towards people who use drugs by key stakeholders appeared to interfere with naloxone administration, which led to unsuccessful outcomes.

#### Sub-theory 1: Low threshold contexts generate responsibility, self-efficacy and willingness, which reduced bystander effects leading to reversals on members within the social network (NBIT-A)

Contexts that have a non-judgemental attitude towards the behaviour and identity of a person who uses drugs foster the environment necessary to make naloxone-based interventions successful. This context generates mechanisms of responsibility which enhances in-group norms of helping someone of the same social category. This generated outcomes of reversals on overdose on friends or acquaintances (outcomes) [43–46]. Specific low threshold contexts such as a syringe exchange programme that employed naloxone-based training generated feelings of self-efficacy (mechanism). Participants reported a belief in their ability to respond to an overdose, mitigating any bystander effects [47]. There were CMOc where mechanisms were unclear, however, were inferred from outcomes. In such cases similar mechanisms assumed to be at work (responsibility and willingness) which led to administration of naloxone to friends of the rescuer, acquaintances, or strangers (outcome) [18, 48, 49].

#### Sub-theory 2: Low threshold contexts generate successful outcomes. This produces a second context of peer-to-peer training, which generates willingness, trust, responsibility and confidence (mechanisms). Those trained by their peers in unofficial training context displays group norms of helping using naloxone to reverse an overdose on someone within their social networks/ shared social category (outcome) (NBIT-B)

This sub-theory builds upon the outcome found in NBIT-A. Whereas the first outcome (a reversal of an overdose on a peer) creates a ripple effect and generated a new context of individuals of the target group (peer-to-peer) who witness naloxone use. A naloxone-based training in a low threshold context generated the mechanisms of willingness (mechanism) to help, trust, and responsibility (as found in NBIT-A). This generated outcomes where a bystander used naloxone to reverse an overdose on a peer. As a peer witness another peer helping a member of the same social category, the witness becomes knowledgeable of the signs and symptoms of overdose, the benefits of using naloxone-based interventions, and the skills to reverse an overdose. A shared social identity/ social category appeared to reduce bystander effects in such circumstances. This context generated willingness, trust, and confidence (mechanism) in one’s ability to administer the naloxone from peer training outside of an official training site and sharing the intervention within their social networks. In-group norms of helping supported the use of naloxone through the process of diffusion. Administration of naloxone occurred by an untrained bystander in an overdose emergency (outcome) [18, 40].

#### Sub-theory 3: Negative attitudes towards people who use opioids by stakeholders (e.g. police, ambulance staff) alongside a low threshold context creates conflict. This generates feelings of stigma, fear and mistrust, leading to lack of support for helping someone in a shared social category. This led to not responding to an overdose emergency or carrying the kit (NBIT-C)

Contexts with a low threshold setting coupled with an environment of negative attitudes towards bystander use of naloxone by stakeholders such as probation officers and doctors and first responders such as police and emergency medical staff were identified. This type of environment appeared to generate feelings of stigma, fear and mistrust and inhibited the bystander response. For example, participants reported police confiscation of the naloxone kit, perceiving it as drug paraphernalia [47]. Medical professionals called to assist in an overdose emergency criticised the bystander’s use of the naloxone, suggesting that they were not fit to use such items [50]. Participants with a legal prescription of naloxone were told they were violating terms of probation when found in their possession [23]. This context does not support the social identity of a person to be an empowered bystander to act in an overdose, as found in NBIT-A and NBIT-B. This context instead generated feelings of stigma and fear (mechanism) which led to a lack of responding in an overdose after being trained to do so [46]. There were also reports of individuals not carrying naloxone on their person, and thus, they could not administer naloxone when needed (outcome) [23].

### Programme theory 2: Skills Transfer Theory (STT)

There were two sub-theories under Skills Transfer Theory (STT)-A and STT-B. Both discuss how a low threshold context generated trust between the trainee with the trainer, confidence, self-efficacy, and willingness to use the skills from the naloxone training (mechanism). This led to the effective use of naloxone, leading to a full reversal (outcome). However, one sub-theory depicts how such a context generated empowerment which led to full reversals (outcomes). In this case, a full reversal led to the perception that additional help (ambulance) was not needed (part of the training in successful naloxone administration). This resulted in a lack of ambulance callouts as an outcome of the intervention training.

#### Sub-theory 1: A low threshold context enhances trust between the trainer and the trainee, generating confidence, self-efficacy, and willingness to use the skills from the naloxone training (mechanism). Effective use of the naloxone leads to a full reversal (outcome) (STT-A)

Naloxone training was placed in a low threshold environment in several evaluations [47, 51, 52]. Such a setting generated feelings of self-efficacy and confidence (mechanism) in using overdose prevention skills. This came from direct reports of participants feeling confident in having naloxone to help, followed by a sense of control, and efficacy knowing what steps to take during an overdose [14, 50]. There were also reports of building capacity to respond resulting in a full reversal (outcome). This context also generated use of skills, such as CPR, placing the victim in a rescue position, and naloxone administration (mechanisms). Participants reported rescue breathing, calling for emergency help, staying with the victim, and multiple naloxone administrations with outcomes leading to full reversals [24, 39, 50, 53, 54].

#### Sub-theory 2: Low threshold contexts generate empowerment, confidence, and use of skills learned in overdose training (mechanisms). This led to successful reversals and skills for post overdose management, such as calling for emergency help after a reversal (outcome), were not reported

Evaluations based on a low threshold needle exchange support this theory in populations pre-exposed to overdose management [22, 53–56]. A low threshold context appeared to generate outcomes where there was a lack of reports of using ambulance services after the in-house training compared to baseline measurements. Participants described mechanisms of confidence in their ability to manage an overdose, leading to the lack of using outside help [53, 54]. This implied the low threshold context generated feelings of empowerment.For example, participants reported that the successful reversal was evidence of full recovery and decided not to call for an ambulance, a part of post overdose management (outcome) [52]. Participants in other literature reported not calling an ambulance “… because the overdose could be managed by themselves alone” (Baca et al., pg. 65) or there were direct reports of a lack of perceived need to call an ambulance [22, 55, 56].

## Discussion

### Summary of the findings

The results of this synthesis showed that a low threshold context is central to reversing an overdose using naloxone. This context appeared to minimise bystander effects and generated mechanisms of willingness, responsibility, confidence, empowerment, and self-efficacy. This led to successful skills transfer and the use of naloxone training by both trained and untrained bystanders on someone within their social networks. Skills transfer also leads to outcomes of successful reversals and lack of calling for emergency help (post overdose management). Outcomes become problematic when the intervention is placed in a low threshold environment alongside negative attitudes towards people who use opioids It can generate mechanisms of fear, stigma, and bystanders may not be willing to reverse an overdose on their peer. This leads to outcomes where the use of naloxone is not reported, and the intervention is unsuccessful.

The review provides a theoretical outline of how out-group normative beliefs—for example people who do not use drugs but have a non-judgemental attitude towards drug use (harm reduction training setting)—appears to reinforce the social identity and in-group norms of helping people who use drugs. The relationship between the setting and outcomes was not explored in systematic reviews involving community-based training contexts [7, 8]. Previous systematic reviews on naloxone have not explained why people who use drugs, their families and friends are suitable trainees for naloxone-based interventions [6–8]. This review has identified how shared social categorical membership of these trainees reduces bystander effects, which leads to a quicker response to prevent an opioid overdose. Other researchers have suggested increased naloxone provision may increase mortality through moral hazard [9]. However, this was not supported by this review. This review instead explained how an increase in overdose mortality can be a matter of negative normative beliefs of the out-group—in this case people not of the same social category of people who use drugs such as first responders. This in turn appeared to shift in-group norms (people who use drugs) of helping people within their own social category. This is evident from results of these studies where naloxone was not used or where there was a lack of carriage resulting in increased mortality outcomes. This review also fills in the gap for those studies not used in this review by outlining how the out-group norms of a low threshold training context supports people to use naloxone [57–60].

### Strengths and limitation of the review

A realist review supplies an understanding of how an intervention works, which can help inform decisions to make it more effective. Overdose prevention is not simply the effective use of naloxone-based interventions; this review excludes other overdose prevention strategies such as medication assisted treatments, limited prescribing and drug consumption rooms or the interactions of these with naloxone-based interventions. Results of a realist review are typically based on a final programme theory and cannot be generalised, as training contexts are unique to their location and availability of training. Although the literature can provide evidence of this theory, it can only infer casual explanations to generalise the effectiveness of naloxone. A realist review can only supply an understanding of the conditions in which the programme may work to produce successful outcomes. In-depth notation of legal contexts related to naloxone, or where other interventions that use naloxone such as within supervised injection sites may be useful for future reviews.

### Recommendations for design and implementation of naloxone into health care

#### Increase low threshold training settings

Training based in a harm reduction approach will be helpful for community pharmacists who counsel clients in naloxone [34]. This will help shift out-group norms, alter attitudes, and perceptions towards people who use drugs. It can create a non-stigmatising environment to get naloxone and/or be trained in naloxone administration and overdose prevention, helping to generate successful outcomes. Police have the potential to aid in the reduction of overdose deaths as a first responder [61–63]. Training based in a non-judgemental approach for typical ‘out-groups’ of those who use drugs can reduce negative attitudes and may alter group norms regarding people who use drugs.

#### Peer-to-peer naloxone trainig

Clinicians, policymakers, and trainers may wish to use this review to inform the design of naloxone-based programmes.. Programmes may wish to use peer-to-peer training and distribution of naloxone as unofficial, peer-to-peer training of naloxone appeared to minimise bystander effects, enhance in-group norms of helping, and aid in distribution of naloxone through social networks. Such programmes are already in place and the evaluations of their effectiveness are forthcoming [64–66There is also a core role in contexts that work for meaningful consultation and working alongside people who use drugs in naloxone-based intervention design, training, and implementation. Naloxone training programmes may incorporate dynamics of helping in groups with emphasis on the process of diffusion of responsibility. This approach can reduce bystander effects and has been suggested for training where bystander intervention is needed, such as CPR [67].

#### Stigma reduction: Good Samaritan laws and stigma campaigns

A major barrier in using naloxone-based interventions was fear of arrest by the police. Laws granting immunity and Good Samaritan laws that protect the bystander from criminal charges from drug-related charges when involved in an opioid overdose have helped to reduce opioid overdose mortality [68, 69]. Good Samaritan laws that have been adopted in 30 States in the United States and in British Columbia, Canada [68, 70]. Having such a law creates an environment of safety to use the training and support group norms of helping, however, not all bystanders are aware of this law and similar mechanisms of stigma and fear continue [69]. Police can have mixed views towards Good Samaritan laws and may continue to respond with negative attitudes towards people at the scene of an overdose [62]. Stigma campaigns that run parallel to changes in the law would be beneficial. For example, communication of sympathetic and positive narratives regarding people who use drugs/opioids, and increase contact with people who use drugs has been found to decrease stigma and may be helpful in training for key stakeholders and first responders [71, 72]. Educating service users on Good Samaritan laws and the social dynamics of helping in these contexts may also reduce fear and poor outcomes.

## Conclusion

This review provides theoretically informed guidance aimed to reduce contextual harms embedded within the social environment associated with using opioid and naloxone implementation [4, 73]. This review evidenced the contexts that work, i.e. low threshold/harm reduction contexts supportive of people who use drugs. It also generated mechanisms where the naloxone-based intervention was successful, noting that; negative attitudes towards the use of naloxone-based interventions and harm reduction and/or a judgmental attitude towards drug use in one social environment may hinder the power of social diffusion. This leads to outcomes where the naloxone-based intervention is not effective. This can be an indirect factor contributing to overdose deaths occurring in these contexts. Researchers and policy makers may wish to identify the public attitudes towards people who use opioids and interventions to prevent overdose deaths to mitigate any implementation problems to ensure harm reduction strategies are effective.

## Supplementary Information

**Additional file 1.** RAMESES checklist for the realist review for naloxone-based interventions.

**Additional file 2.** Identification and selection tools for the realist review of naloxone-based interventions.

**Additional file 3.** Appraisal and rigor tools for the realist review of naloxone-based interventions.

**Additional file 4.** Data extraction and synthesis tools for the realist review of naloxone-based interventions

**Additional file 5.** References for the realist review of naloxone-based interventions

## Data Availability

All data generated or analysed during this study are included in this published article [and its supplementary information files]. Datasets that are not a part of this supplementary file can be made available from the corresponding author on reasonable request.

## References

[1] Ritchie H, Roser M. Drug use. 2020. https://ourworldindata.org/drug-use.com. Accessed 4 May 2021.

[2] Strang J, Manning V, Mayet S (2008). Overdose training and take-home naloxone for opiate users: prospective cohort study of impact on knowledge and attitudes and subsequent management of overdoses. Addiction.

[3] Doe-Simkins M, Walley AY, Epstein A (2009). Saved by the nose: bystander-administered intranasal naloxone hydrochloride for opioid overdose. AM J Public Health.

[4] Craig P, Di Ruggiero E, Frolich KL (2018). Taking account of context in population health intervention research: guidance for producers, users and funders of research.

[5] McAuley A, Aucott L, Matheson C (2015). Exploring the life-saving potential of naloxone: a systematic review and descriptive meta-analysis of take home naloxone (THN) programmes for opioid users. Int J Drug Policy.

[6] McDonald R, Strang J (2016). Are take-home naloxone programmes effective? Systematic review utilizing application of the Bradford Hill criteria. Addiction.

[7] Mueller SR, Walley AY, Calcaterra SL (2015). A review of opioid overdose prevention and naloxone prescribing: implications for translating community programming into clinical practice. Subst Abuse.

[8] Clark AK, Wilder CM, Winstanley EL (2014). A systematic review of community opioid overdose prevention and naloxone distribution programs. J Addict Med.

[9] Doleac JL, Mukherjee A. The moral hazard of lifesaving innovations: naloxone access, opioid abuse, and crime. 2018. https://ssrn.com/abstract=3170278. Accessed 5 Mar 2021.

[10] Ndrianasy E. The opioid crisis: naloxone access laws and moral hazard. PhD [dissertation]. Tennessee: Middle Tennessee State University; 2019. https://jewlscholar.mtsu.edu/bitstream/handle/mtsu/6085/Ndrianasy_mtsu_0170E_11203.pdf?sequence=1&isAllowed=y.

[11] Wong G, Westhorp G, Pawson R, et al. Realist synthesis: rameses training materials. 2013. https://www.ramesesproject.org/media/Realist_reviews_training_materials.pdf. Accessed 5 Sept 2019.

[12] Pawson R (2006). Evidence based policy: a realist perspective.

[13] Pawson R, Greenhalgh T, Harvey G (2005). Realist review-a new method of systematic review designed for complex policy interventions. J Heath Serv Res Pol.

[14] Banjo O, Tzemis D, Al-Qutub D (2014). A quantitative and qualitative evaluation of the British Columbia Take Home Naloxone program. CMAJ Open.

[15] Bennett T, Holloway K (2012). The impact of take-home naloxone distribution and training on opiate overdose knowledge and response: an evaluation of the THN Project in Wales. Drugs Edu Prev Pol.

[16] Clarke A, Eustace A. Evaluation of the HSE Naloxone Demonstration Project. 2016. https://www.drugsandalcohol.ie/26037/1/Naloxonedemoproject.pdf. Accessed 5 Sept 2019.

[17] Dettmer K, Saunders B, Strang J (2001). Take home naloxone and the prevention of deaths from opiate overdose: two pilot schemes. BMJ.

[18] Doe-Simkins M, Quinn E, Xuan Z (2014). Overdose rescues by trained and untrained participants and change in opioid use among substance-using participants in overdose education and naloxone distribution programs: a retrospective cohort study. BMC Public Health.

[19] Dwyer K, Walley AY, Langlois BK (2015). Opioid education and nasal naloxone rescue kits in the emergency department. WestJem.

[20] Leece PN, Hopkins S, Marshall C (2013). Development and implementation of an opioid overdose prevention and response program in Toronto, Ontario. Can J Public Health.

[21] McAuley A, Lindsay G, Woods M (2010). Responsible management and use of a personal take-home naloxone supply: a pilot project. Drugs Educ Prev Pol.

[22] Seal KH, Thawley R, Gee L (2005). Naloxone distribution and cardiopulmonary resuscitation training for injection drug users to prevent heroin overdose death: a pilot intervention study. J Urban Health.

[23] Shorter G, Bingham T. Service review: take home Naloxone programme in NI consultation with service users and service providers. 2016. https://www.drugsandalcohol.ie/25353/1/PHANI_Naloxone-service-evaluation-final-report.pdf. Accessed 1 June 2019.

[24] Wagner KD, Valente TW, Casanova M (2010). Evaluation of an overdose prevention and response training programme for injection drug users in the Skid Row area of Los Angeles, CA. Int J Drug Policy.

[25] Walley AY, Xuan Z, Hackman HH (2013). Opioid overdose rates and implementation of overdose education and nasal naloxone distribution in Massachusetts: interrupted time series analysis. BMJ.

[26] Latane B, Darley J (1968). Group inhibition of bystander intervention in emergencies. J Pers Soc Psychol.

[27] Levine M, Cassidy C, Stürmer S, Snyder M (2009). Groups, identities, and bystander behavior. The psychology of prosocial behavior group processes, intergroup relations, and helping.

[28] Kraiger K, Passmore J, Dos Santos N, Kraiger K, Passmore J, Dos Santos N, Malvezz S (2014). The psychology of training, development, and performance improvement. The Wiley Blackwell handbook of the psychology of training development and performance improvement.

[29] Orfaly R, Frances J, Campbell P (2005). Train-the-trainer as an educational model in public health preparedness. JPHMP.

[30] Veri CC, Vonder Haar TA (1971). Training the trainer.

[31] Chimbar L, Moleta Y (2018). Naloxone effectiveness: a systematic review. J Addict Nurs.

[32] European Monitoring Centre for Drugs and Drug Addiction. Preventing fatal overdoses: a systematic review of the effectiveness of take-home naloxone. 2015. https://www.emcdda.europa.eu/publications/emcdda-papers/naloxone-effectiveness_en. Accessed 5 Sept 2019.

[33] Horton M, McDonald R, Green TC (2017). A mapping review of take-home naloxone for people released from correctional settings. Int J Drug Policy.

[34] Nielsen S, Van Hout MC (2016). What is known about community pharmacy supply of naloxone? A scoping review. Int J Drug Policy.

[35] Willman MW, Liss DB, Schwarz ES (2017). Do heroin overdose patients require observation after receiving naloxone?. Clin Toxicol.

[36] Ruane L. The overdose drug Naloxone can save lives so let's remove the barriers to accessing it. The Journal IE [Internet]. 2019 April 14. Cited 5 Sept 20. https://www.thejournal.ie/readme/lynn-ruane-naloxone-can-save-the-life-of-people-who-overdose-lets-remove-all-barriers-to-access-4589304-Apr2019/.

[37] Public health agency. Take Home Naloxone Report on supply and use to reverse an overdose. 2012-2016. 2016. https://www.publichealth.hscni.net/sites/default/files/Take%20Home%20Naloxone%20Report%202012-16_0.pdf. Accessed 5 Sept 2019

[38] Center for Disease Control. National Vital Statistics Rapid Release Provisional Drug Overdose Death Counts. 2021. https://www.cdc.gov/nchs/nvss/vsrr/drug-overdose-data.htm. Accessed 2 Jan 2021.

[39] National Institute for Health development. Opioids overdose deaths prevention programme in Estonia. https://intra.tai.ee//images/prints/documents/154651154294_NaloksoonEestis_eng.pdf. Accessed 5 June 2019.

[40] Bowles JM, Lankenau SE (2019). “I gotta go with modern technology, so i’m gonna give ’em the Narcan”: the diffusion of innovations and an opioid overdose prevention program. Qual Health Res.

[41] Sherman SG, Gann DS, Tobin KE (2009). “The life they save may be mine”: diffusion of overdose prevention information from a city sponsored programme. Int J Drug Policy.

[42] Jagosh J, Bush PL, Salsberg J (2015). A realist evaluation of community-based participatory research: partnership synergy, trust building and related ripple effects. BMC Public Health.

[43] George S, Boulay S, Begley D (2010). I saved a life”: a heroin addict’s reflections on managing an overdose using “take home naloxone. BMJ Case Rep.

[44] Maxwell S, Bigg D, Stanczykiewicz K (2006). Prescribing naloxone to actively injecting heroin users: a program to reduce heroin overdose deaths. J Addict Dis.

[45] Olsen A, McDonald D, Lenton S, et al. Independent evaluation of the ‘Implementing Expanded Naloxone Availability in the ACT (I-ENAACT)’Program, 2011–2014 Final report. Canberra: ACT Health. 2015. Updated 2015 August; cited 5 Sept 2019. https://www.health.act.gov.au/sites/default/files/2018-09/Naloxone%20Evaluation%20Report_Aug_2015.pdf.

[46] Worthington N, Piper TM, Galea S (2006). Opiate users’ knowledge about overdose prevention and naloxone in New York City: a focus group study. Harm Reduct J.

[47] Lankenau SE, Wagner KD, Silva K (2013). Injection drug users trained by overdose prevention programs: responses to witnessed overdose. J Community Health.

[48] Rowe C, Santos GM, Vittinghoff E (2015). Predictors of participant engagement and naloxone utilization in a community-based naloxone distribution program. Addiction.

[49] Siegler A, Huxley-Reicher Z, Maldjian L (2017). Naloxone use among overdose prevention trainees in New York City: a longitudinal cohort study. Drug Alcohol Depend.

[50] Sherman SG, Gann DS, Scott G (2008). A qualitative study of overdose responses among Chicago IDUs. Harm Reduct J.

[51] Nelson M, Lenton S, Dietze P, et al. Evaluation of the WA peer naloxone project–final report. Report for Western Australia: National Drug Research Institute, Perth. 2016. https://www.researchgate.net/profile/Anna_Olsen/publication/307605397_Evaluation_of_the_WA_Peer_Naloxone_Project_Final_Report/links/58ce0370aca27233551623db/Evaluation-of-the-WA-Peer-Naloxone-Project-Final-Report.pdf. Accessed 5 Sept 2019.

[52] Wagner KD, Davidson PJ, Iverson E (2014). “I felt like a superhero”: the experience of responding to drug overdose among individuals trained in overdose prevention. Int J Drug Policy.

[53] Bennett AS, Bell A, Tomedi L (2011). Characteristics of an overdose prevention, response, and naloxone distribution program in Pittsburgh and Allegheny County, Pennsylvania. J Urban Health.

[54] Maldjian L, Siegler A, Kunins HV (2016). Evaluation of overdose prevention trainings in New York City: knowledge and self- efficacy among participants 12 months after training. Subst Abuse.

[55] Tobin KE, Sherman SG, Beilenson P (2009). Evaluation of the Staying Alive programme: training injection drug users to properly administer naloxone and save lives. Int J Drug Policy.

[56] Baca CT, Grant KJ (2007). What heroin users tell us about overdose. J Addict Dis.

[57] Albert S, Brason FW, Sanford CK (2011). Project Lazarus: community-based overdose prevention in rural North Carolina. Pain Med.

[58] Alexandridis AA, McCort A, Ringwalt CL (2018). A state-wide evaluation of seven strategies to reduce opioid overdose in North Carolina. Inj Prev.

[59] Lagu T, Anderson BJ, Stein M (2006). Overdoses among friends: drug users are willing to administer naloxone to others. J Subst Abuse Treat.

[60] Lewis DA, Park JN, Vail L (2016). Evaluation of the overdose education and naloxone distribution program of the Baltimore student harm reduction coalition. Am J Public Health.

[61] Rando J, Broering D, Olson JE (2015). Intranasal naloxone administration by police first responders is associated with decreased opioid overdose deaths. Am J Emerg Med.

[62] Wagner KD, Bovet LJ, Haynes B (2016). Training law enforcement to respond to opioid overdose with naloxone: impact on knowledge, attitudes, and interactions with community members. Drug Alcohol Depend.

[63] Davis CS, Carr D, Southwell JK (2015). Engaging law enforcement in overdose reversal initiatives: authorization and liability for naloxone administration. Am J Public Health.

[64] European network of people who use drugs. Peer to Peer distribution of naloxone (P2PN). 2019. https://www.euronpud.net/naloxone. Accessed 5 May 2020

[65] Marshall C, Perreault M, Archambault L (2017). Experiences of peer-trainers in a take-home naloxone program: results from a qualitative study. Int J Drug Policy.

[66] Waye KM, Goyer J, Dettor D (2019). Implementing peer recovery services for overdose prevention in Rhode Island: an examination of two outreach-based approaches. Addict Behav.

[67] Vaillancourt C, Stiell IG, Wells GA (2008). Understanding and improving low bystander CPR rates: a systematic review of the literature. CJEM.

[68] McClellan C, Lambdin BH, Ali MM (2018). Opioid-overdose laws association with opioid use and overdose mortality. Addict Behav.

[69] Schneider KE, Park JN, Allen ST (2020). Knowledge of good samaritan laws and beliefs about arrests among persons who inject drugs a year after policy change in Baltimore, Maryland. Public Health Rep.

[70] Annual Statutes-Good Samaritan Drug Overdose Act 2017 (Canada) S.C.2017. c.4. https://laws.justice.gc.ca/eng/AnnualStatutes/2017_4/page-1.html.

[71] Bachhuber MA, McGinty EE, Kennedy-Hendricks A (2015). Messaging to increase public support for naloxone distribution policies in the United States: results from a randomized survey experiment. PLoS ONE.

[72] Livingston JD, Milne T, Fang ML (2012). The effectiveness of interventions for reducing stigma related to substance use disorders: a systematic review. Addiction.

[73] Rhodes T (2009). Risk environments and drug harms: a social science for harm reduction approach. Int J Drug Policy.

