# Supplementary material for: How do naloxone-based interventions work to reduce overdose deaths: a realist review
Source: Harm Reduct J. 2022 Feb 23;19:18. doi: 10.1186/s12954-022-00599-4 (PMC8867850; doi:10.1186/s12954-022-00599-4)
Supplement: Supplementary file 2 — Additional file 2. Identification and selection tools for the realist review of naloxone-based interventions. [file 12954_2022_599_MOESM2_ESM.docx]

**Additional file 2: Identification and selection tools for the realist review of naloxone based interventions**

| **Identification tool (Rated on a scale, Yes= 1, No= 0, Unsure=2)** | | | |
| --- | --- | --- | --- |
|  | Yes | No | Unsure |
| 1. Is this study a pilot, feasibility, evaluation, randomized control, quasi experimental, case study and or a qualitative work? |  |  |  |
| 1. Does the literature include populations of 17 years or older, those who are abstinent, non-abstinent and have a history of opioid use? |  |  |  |
| 1. Does the literature describe an opioid overdose due to prescribed opioid medication (medical and non-medical prescription), illicit opioids such as heroin, opioid synthetics such as fentanyl, and polysubstance use that includes opioids/ opiates? |  |  |  |
| 1. Does the literature discuss an intervention to reduce overdose deaths in which naloxone is a component? |  |  |  |
| 1. Does the literature contain populations of any sex or gender? |  |  |  |
| 1. Is the literature from any country? |  |  |  |
| 1. Is the literature written in the English language? |  |  |  |
| **Retain the article?** | | | |

| **Selection tool (Yes=1, No=0. Items is retained if it has a score of 2 or more)** | | | |
| --- | --- | --- | --- |
|  | **Yes** | **No** | **Total** |
| 1. Does the literature report reversals of opioid related overdose use a naloxone-based intervention? |  |  |  |
| 1. Does the literature report the situation of the reversal such as the environment and for whom it was administered? |  |  |  |
| 1. Does the literature report training in the use of a naloxone-based intervention? |  |  |  |
| 1. Does the literature report the direct experience of administration of a naloxone-based intervention? For examples actions taken to manage the overdose? |  |  |  |
| **Total:** | | | |
