# Supplementary material for: How do naloxone-based interventions work to reduce overdose deaths: a realist review
Source: Harm Reduct J. 2022 Feb 23;19:18. doi: 10.1186/s12954-022-00599-4 (PMC8867850; doi:10.1186/s12954-022-00599-4)
Supplement: Supplementary file 3 — Additional file 3. Appraisal and rigor tools for the realist review of naloxone-based interventions. [file 12954_2022_599_MOESM3_ESM.docx]

**Additional file 3: Appraisal and rigor tools for the realist review of naloxone based interventions**

| **Appraisal based on relevance**  Papers that answer yes to question 1 and provide one or more aspects of a CMOc are retained. Rating for Relevance (1=High to 4=Low). 1= Research aim and research questions are matched to the RR question. Findings are clearly described, good descriptions of CMOc are found which can help move the theory forward. All others decrease in relevance to the RR (Lowest 4). Literature that has no CMOc are excluded. | | | |
| --- | --- | --- | --- |
|  | **YES** | **NO** | **TOTAL** |
| 1. Do the aims of the study adequately match the aims/ research question of the review? |  |  |  |
| 1. Does the paper describe a full, partial context in which the intervention training? |  |  |  |
| 1. If so, how many parts of the context are covered? (Place the total amount)   **Context ex:**   - Institutional- Rules related to the programme as set out in government policy - Infrastructure- Cultural setting of the programme - Interpersonal-Relationship between stakeholders - Individuals- Characteristics of the stakeholders involved | | |  |
| 1. Does the paper describe explicit/ direct and implicit/ indirect outcomes relevant to the intervention in detail? If so, is there more than one?   Outcomes ex:   - Quantitative outcomes from pre and post tests - Quantitative frequency of use of overdose management skills - Subjective reports from intervention populations |  |  |  |
| 1. Does the paper provide insight into mechanisms? |  |  |  |
| 1. If so, how many mechanisms were identified? (Place the total amount) |  | |  |
| **Total amount of possible configurations** | | |  |
| **Rating for relevance (1 High- 4 Low)** | | |  |

| **Rigor** | | |
| --- | --- | --- |
|  | YES | NO |
| Does the paper describe the methodology? |  |  |
| Does the paper describe data collection methods? |  |  |
| Does the paper describe analysis methods? |  |  |
| Is the methodology congruent with the reported outcomes? |  |  |
| Are there limitations to the methods and its relationships to outcomes? |  |  |
