# Supplementary material for: How do naloxone-based interventions work to reduce overdose deaths: a realist review
Source: Harm Reduct J. 2022 Feb 23;19:18. doi: 10.1186/s12954-022-00599-4 (PMC8867850; doi:10.1186/s12954-022-00599-4)
Supplement: Supplementary file 4 — Additional file 4. Data extraction and synthesis tools for the realist review of naloxone-based interventions [file 12954_2022_599_MOESM4_ESM.docx]

**Additional file 4. Data extraction and synthesis tools for the realist review of naloxone-based interventions**

| **Data Extraction** | | | | | | |
| --- | --- | --- | --- | --- | --- | --- |
|  |  | | |  | | |
| **Study #** | **Study Characteristics** | **YES** | **NO** |  | | |
|  | 1. Citation |  |  |  |  |  |
|  | 1. Times cited |  |  |  |  |  |
|  | 1. Date |  |  |  |  |  |
|  | 1. Country/ State (if relevant) |  |  |  |  |  |
|  | 1. Type of study |  |  |  |  |  |
|  | 1. Methods: How data was collected, how long data was collected and recruitment |  |  |  |  |  |
|  | 1. Population/ Sample |  |  |  |  |  |
|  | 1. TiDier Checklist |  |  |  |  |  |
|  | 1. Outcomes and results |  |  |  |  |  |
|  | 1. Strengths and weaknesses |  |  |  |  |  |
|  | |  | | | | |
| **Synthesis** | |  |  |  | | |
|  | |  |  | **Hypothesis** | | |
| **Study #** | **Codes** | **YES** | **NO** | **A** | **B** | **C** |
|  | 1. Programme theory 1- Blue |  |  |  |  |  |
|  | 1. Programme theory 2-Purple |  |  |  |  |  |
|  | 1. New programme theory- Pink |  |  |  |  |  |
|  | 1. Context-Yellow |  |  |  |  |  |
|  | 1. Mechanism- Green |  |  |  |  |  |
|  | 1. Outcomes- Orange |  |  |  |  |  |
|  | 1. CMOc were configured |  |  |  |  |  |
|  | 1. Hypothesis were refuted or supported |  |  |  |  |  |
