# Supplementary material for: How do naloxone-based interventions work to reduce overdose deaths: a realist review
Source: Harm Reduct J. 2022 Feb 23;19:18. doi: 10.1186/s12954-022-00599-4 (PMC8867850; doi:10.1186/s12954-022-00599-4)
Supplement: Supplementary file 5 — Additional file 5. References for the realist review of naloxone-based interventions [file 12954_2022_599_MOESM5_ESM.docx]

**Table 1 References**

1. Ambrose G, Amlani A, and Buxton JA. Predictors of seeking emergency medical help during overdose events in a provincial naloxone distribution programme: A retrospective analysis. *BMJ Open* 2016; *6*: 1–8. doi.org/10.1136/bmjopen-2016-011224.
2. Baca CT and Grant KJ. What heroin users tell us about overdose. *J Addict D* 2007; *26*: 63–68. doi.org/10.1300/J069v26n04_08
3. Banjo O, Tzemis D, Al-Qutub D, et al. A quantitative and qualitative evaluation of the British Columbia Take Home Naloxone program. *CMAJ Open* 2014; *2*: E153–E161. doi.org/10.9778/cmajo.20140008
4. Behar E, Santos GM, Wheeler E, et al. Brief overdose education is sufficient for naloxone distribution to opioid users. *Drug Alcohol Depend* 2015; *148*: 209–212. doi.org/10.1016/j.drugalcdep.2014.12.009
5. Bennett AS, Bell A, Tomedi L, et al. Characteristics of an overdose prevention, response, and naloxone distribution program in Pittsburgh and Allegheny County, Pennsylvania. *J. Urban Health* 2011; *88*: 1020–1030. doi.org/10.1007/s11524-011-9600-7.
6. Bennett T, and Holloway K. The impact of take-home naloxone distribution and training on opiate overdose knowledge and response: An evaluation of the THN Project in Wales. *Drugs* *Edu Prev Pol* 2012; *19*: 320–328. doi.org/10.3109/09687637.2012.658104
7. Beswick T, Best D, Bearn J, et al. From salt injection to naloxone: accuracy and myths in peer resuscitation methods for opiate overdose. *J. Drug Issues* 2002; *3*:1103-1114.
8. Bowles JM, and Lankenau, SE. “I Gotta Go With Modern Technology, So I’m Gonna Give ’em the Narcan”: The Diffusion of Innovations and an Opioid Overdose Prevention Program. *Qual. Health Res* 2019; *29*: 345–356. doi.org/10.1177/1049732318800289
9. Clarke A and Eustace A. Evaluation of the HSE Naloxone Demonstration Project. https://www.drugsandalcohol.ie/26037/1/Naloxonedemoproject.pdf (2016, Accessed 5, September 2019)
10. Chronister KJ, Lintzeris N, Jackson A, et al. Findings and lessons learnt from implementing Australia’s first health service based take-home naloxone program. *Drug Alcohol Rev* 2018; *37*: 464–471. doi.org/10.1111/dar.12400.
11. Das S, Shah N, and Ghadiali M. Intravenous use of intranasal naloxone: A case of overdose reversal. *Subst Abus* 2017; *38*:18–21. doi.org/10.1080/08897077.2016.1267686.
12. Dettmer K, Saunders B, Strang J. Take home naloxone and the prevention of deaths from opiate overdose: two pilot schemes. *BMJ* 2001; 322: 895-6.
13. Doe-Simkins M, Walley AY, Epstein A, Moyer P. Saved by the nose: bystander-administered intranasal naloxone hydrochloride for opioid overdose. *AM J Public Health*. 2009; 99: 788-91.
14. Doe-Simkins M, Quinn E, Xuan Z, et al. Overdose rescues by trained and untrained participants and change in opioid use among substance-using participants in overdose education and naloxone distribution programs: A retrospective cohort study. *BMC Public Health* 2014; *14*. doi.org/10.1186/1471-2458-14-297.
15. Dwyer K, Walley AY, Langlois BK, et al. Opioid education and nasal naloxone rescue kits in the emergency department. *WestJem* 2015;16: 381.
16. Enteen L, Bauer J, McLean R, et al. Overdose prevention and naloxone prescription for opioid users in San Francisco. *J Urban Health* 2010; *87*: 931–941. doi.org/10.1007/s11524-010-9495-8.
17. Espelt A, Bosque-Prous M, Folch C, et al. Is systematic training in opioid overdose prevention effective? *PLoS ONE* 2107; *12*: 1–14. doi.org/10.1371/journal.pone.0186833
18. European network of people who use drugs. *Peer to Peer distribution of naloxone (P2PN).* (2019, accessed 5 May 2020) https://www.euronpud.net/naloxone.
19. Galea S, Worthington N, Piper T, et al. Provision of naloxone to injection drug users as an overdose prevention strategy: Early evidence from a pilot study in New York City. *Addict Behav* 2006; *31*: 907–912. doi.org/10.1016/j.addbeh.2005.07.020.
20. Gaston RL, Best D, Manning V, et al. Can we prevent drug related deaths by training opioid users to recognise and manage overdoses? *Harm Reduct J 2009*; *6*-26. doi.org/10.1186/1477-7517-6-26
21. George S, Boulay S, and Begley D. ‘I saved a life’: A heroin addict’s reflections on managing an overdose using ‘take home naloxone’ *BMJ Case Repor* 2010; 1–4. doi.org/10.1136/bcr.05.2010.2986.
22. Gilbert L, Hunt T, Primbetova S, et al. Reducing opioid overdose in Kazakhstan: A randomized controlled trial of a couple-based integrated HIV/HCV and overdose prevention intervention “Renaissance.” *Inter J Drug Policy*, 2018; *54*: 105–113. doi.org/10.1016/j.drugpo.2018.01.004.
23. Green TC. Distinguishing signs of opioid overdose. *Bone*, 2008; *23*: 1–7. doi.org/10.1038/jid.2014.371.
24. Green TC, Ray M, Bowman SE, et al. Two cases of intranasal naloxone self-administration in opioid overdose. *Subst Abus* 2014; *35*: 129–132. doi.org/10.1080/08897077.2013.825691.
25. Farrugia A, Fraser S, Dwyer R, et al. Take-home naloxone and the politics of care. *Sociol Healh Illn* 2019; *41*: 427–443. doi.org/10.1111/1467-9566.1284.
26. Khatiwoda P, Proeschold-Bell RJ, Meade CS, et al. (2018). Facilitators and Barriers to Naloxone Kit Use Among Opioid-Dependent Patients Enrolled in Medication Assisted Therapy Clinics in North Carolina. *NC Med J* 2018; *79*: 149–155. doi.org/10.18043/ncm.79.3.149.
27. Lankenau SE, Wagner KD, Silva K et al. Injection drug users trained by overdose prevention programs: Responses to witnessed overdose. *J Community Health* 2013; 39: 133-141. doi:10.1007/s10900-012-9591-7
28. Leece PN, Hopkins S, Marshall C, et al. Development and implementation of an opioid overdose prevention and response program in Toronto, Ontario. *Can J. Public Health* 2013; 104: e200-4.
29. Leece P, Gassanov M, Hopkins S, et al. Process evaluation of the Prevent Overdose in Toronto (POINT) program. *C J. Public Health*, 2016; *107*: e224–e230. doi.org/10.17269/CJPH.107.5480.
30. Madah-Amiri D, Clausen T, and Lobmaier P. Rapid widespread distribution of intranasal naloxone for overdose prevention. *Drug and Alcohol Dependence* 2017; *173*: 17–23. doi.org/10.1016/j.drugalcdep.2016.12.013.
31. Madah‐Amiri D, Gjersing L, and Clausen T. Naloxone distribution and possession following a large‐scale naloxone programme. *Addiction*, 2019; *114*: 92-100.
32. Maldjian L, Siegler A, and Kunins, HV. Evaluation of overdose prevention trainings in New York City: Knowledge and self- efficacy among participants 12 months after training. *Subst Abus* 2016; *37*: 459-465. doi.org/10.1080/08897077.2015.1135850.
33. Maxwell S, Bigg D, Stanczykiewicz K et al. Prescribing naloxone to actively injecting heroin users: a program to reduce heroin overdose deaths. *J Addict. Dis* 2006; *25*: 89-96.
34. McAuley A, Lindsay G, Woods M et al. Responsible management and use of a personal take-home naloxone supply: A pilot project. *Drugs Educ Prev Pol* 2010; *17*: 388–399.
35. Nelson M, Lenton S, Dietze P, et al. *Evaluation of the WA peer naloxone project–final report*. Report for Western Australia: National Drug Research Institute, Perth. *(*2016, accessed 5 September 2019*)*
36. National Institute for Health development. Opioids overdose deaths prevention programme in Estonia. Available from: https://intra.tai.ee//images/prints/documents/154651154294_NaloksoonEestis_eng.pdf

(n.d., accessed 5 September 2019)

1. Olsen A, McDonald D, Lenton S, et al. Independent evaluation of the ‘Implementing Expanded Naloxone Availability in the ACT (I-ENAACT)’Program, 2011–2014 Final report. Canberra: ACT Health. 2015. ( updated 2015 August; cited 2019 September 5) <https://www.health.act.gov.au/sites/default/files/2018-09/Naloxone%20Evaluation%20Report_Aug_2015.pdf>
2. Public Health Agency. Take Home Naloxone Report on supply and use to reverse an overdose 2012-2016. Available from: https://www.publichealth.hscni.net/sites/default/files/Take%20Home%20Naloxone%20Report%202012-16_0.pdf. (2016, accessed 5 September 2019)
3. Parmar MKB, Strang J, Choo L, et al. Randomized controlled pilot trial of naloxone-on-release to prevent post-prison opioid overdose deaths. *Addiction* 2017; *112*: 502–515doi.org/10.1111/add.13668
4. Piper TM, Stancliff S, Rudenstine S, et al. Evaluation of a naloxone distribution and administration program in New York City. *Subst. Use Misuse* 2008; *43*: 858-870.
5. Rowe C, Santos, GM, Vittinghoff E, et al. Predictors of participant engagement and naloxone utilization in a community-based naloxone distribution program. *Addiction* 2015: 110: 1301-1310. doi.org/10.1111/add.12961
6. Ruane, L. The overdose drug Naloxone can save lives so let's remove the barriers to accessing it. The Journal IE [Internet]. 2019 April 14, [cited 5 September 20] Available from: https://www.thejournal.ie/readme/lynn-ruane-naloxone-can-save-the-life-of-people-who-overdose-lets-remove-all-barriers-to-access-4589304-Apr2019/
7. Seal, KH, Thawley R, Gee L, et al. Naloxone distribution and cardiopulmonary resuscitation training for injection drug users to prevent heroin overdose death: A pilot intervention study. *J Urban Health* 2005; *82*: 303–311. doi.org/10.1093/jurban/jti053
8. Sherman SG, Gann DS, Scott G, et al. A qualitative study of overdose responses among Chicago IDUs. *Harm Reduct. J*  2008; *5*: 1–5. doi.org/10.1186/1477-7517-5-2.
9. Sherman SG, Gann DS, Tobin, KE,et al. “The life they save may be mine”: Diffusion of overdose prevention information from a city sponsored programme. *Int J Drug Policy* 2009; *20*: 137–142. doi.org/10.1016/j.drugpo.2008.02.004
10. Shorter G, and Bingham T. Service Review: Take Home Naloxone programme in NI Consultation with service users and service providers. Available from: https://www.drugsandalcohol.ie/25353/1/PHANI_Naloxone-service-evaluation-final-report.pdf (2016, accessed 1 June 2019)
11. Siegler A, Huxley-Reicher Z, Maldjian L, et al. Naloxone use among overdose prevention trainees in New York City: A longitudinal cohort study. *Drug Alcohol Depend* 2017; *179*: 124–130. doi.org/10.1016/j.drugalcdep.2017.06.029.
12. Strang J, Manning V, Mayet S, et al. Overdose training and take‐home naloxone for opiate users: prospective cohort study of impact on knowledge and attitudes and subsequent management of overdoses. *Addiction*. 2008;103: 1648-57.
13. Tobin KE, Sherman SG, Beilenson P, et al. Evaluation of the Staying Alive programme: Training injection drug users to properly administer naloxone and save lives. *Int J Drug Policy* 2009; 20: 131-136. doi.org/10.1016/j.drugpo.2008.03.002.
14. Traynor, K. Pharmacist brings naloxone training to her community, *A J Health Syst Pharm* 2019; 76: 1711 doi.org/10.1093/ajhp/zxz199.
15. Wagner KD, Valente TW, Casanova M, et al. Evaluation of an overdose prevention and response training programme for injection drug users in the Skid Row area of Los Angeles, CA. *Int J Drug Policy* 2010; *21*: 186–193.
16. Wagner KD, Davidson PJ, Iverson E, et al. “I felt like a superhero”: The experience of responding to drug overdose among individuals trained in overdose prevention. *Int J Drug Policy* 2014; *25*:157–165. doi.org/10.1016/j.drugpo.2013.07.003.
17. Walley AY, Xuan Z, Hackman HH, et al. Opioid overdose rates and implementation of overdose education and nasal naloxone distribution in Massachusetts: interrupted time series analysis. *BMJ* 2013; 346:f-174. doi.org/10.1136/bmj.f174
18. Worthington N, Piper TM, Galea S, et al. Opiate users’ knowledge about overdose prevention and naloxone in New York City: A focus group study. *Harm Reduct.* J 2006; *3*: 1–7. doi.org/10.1186/1477-7517-3-19
19. Yokell MA, Green TC, Bowman S, et al. Opioid overdose prevention and naloxone distribution in Rhode Island. *Med Health R I*2011; *94*: 240–2
